# Assessing the Speciation of a Cold Water Species, Japanese Sand Lance *Ammodytes personatus*, in the Northwestern Pacific by AFLP Markers

**DOI:** 10.3390/ani8120224

**Published:** 2018-11-28

**Authors:** Zhiqiang Han, Zhiyong Wang, Tianxiang Gao, Takashi Yanagimoto, Koji Iida

**Affiliations:** 1 Fishery College, Zhejiang Ocean University, Zhoushan 316022, China; d6339124@163.com; 2 Fishery College, Jimei University, Xiamen 361021, China; zywang@jmu.edu.cn; 3 National Research Institute of Fisheries Science, Yokohama 236-8648, Japan; yanagimo@fra.affrc.go.jp; 4 Faculty of Fisheries, Hokkaido University, Hakodate 041-8611, Japan; iidacs@fish.hokudai.ac.jp

**Keywords:** *Ammodytes personatus*, climate change, ocean current, sea temperature, Pleistocene glaciation isolation, local adaptation, isolation by distance

## Abstract

**Simple Summary:**

Assessing the status of multiple highly divergent mitochondrial DNA (mtDNA) lineages and delimiting the evolutionarily significant units (ESUs) are the foundation of forecasting the influence of climate change on intraspecific genetic variation. In this study, we employed AFLP to investigate the genetic structure of *Ammodytes personatus* and compared the genetic variation of *A. personatus* in different ocean current systems. The incongruence between nuclear clades and previous mitochondrial lineages suggested that *A. personatus* is indeed composed of at least two genetically divergent cryptic species. Our results demonstrate that intra-species diversity should be taken into account to assess the influence of climate change on species. This study also highlights the value of the natural physical setting created by warm and cold ocean currents in eliciting a correlation between temperature and species distribution. In future studies, the exact range of temperature in both groups must be assessed with a sufficient number of samples to predict the influences of global climate change on both groups.

**Abstract:**

The use of molecular techniques in biodiversity research increasingly results in the recognition of multiple divergent mitochondrial DNA (mtDNA) lineages below the morphospecies level. However, the overlapping distribution of multiple divergent lineages raises the question of whether some of these lineages are in fact cryptic species. Assessing the status of these divergent lineages, delimiting evolutionarily significant units (ESUs), and identifying the dominant evolutionary and ecological drivers are critical components of successful wildlife conservation and management strategies. Amplified fragment length polymorphism (AFLP) markers were applied to characterize the phylogeography pattern of a cold water species, the Japanese sand lance *Ammodytes personatus*, in warm and cold ocean currents. A total of 211 individuals sampled from 12 populations through the species’ range, including samples from Kuroshio Current, Oyashio Current, Tsushima Current, and Yellow Sea, were analyzed. The Bayesian assignment probability test and Neighbor joining (NJ) analysis divided these populations into two genetically and geographically distinct clades (northern and southern clades) characterized by different sea surface temperatures. The incongruence between nuclear clades and previous mitochondrial lineages suggested that *A. personatus* is indeed composed of at least two genetically divergent cryptic species. Pleistocene glaciation isolation after secondary contact, local thermal adaptation, and isolation by distance may explain the observed geographic pattern of two cryptic species and genetic structure within clades.

## 1. Introduction

In evaluating the effects of global climate change on biodiversity, the species was most commonly considered to be the basic unit, and the influences of climate change on genetic variation at the population level were often ignored [1,2,3,4,5]. Pauls et al. highlighted genetic diversity as an important aspect of biodiversity in global climate change studies [1]. Intraspecific genetic variation provides the basis for evolutionary change and is thus the most fundamental level of biodiversity [6,7]. The exploration of the effects of climatic alterations on intraspecific genetic diversity will facilitate the full understanding of the evolutionary consequences of global climate change and its long-term effects on biodiversity [1]. The use of molecular techniques in biodiversity research increasingly results in the recognition of multiple divergent mitochondrial DNA (mtDNA) lineages below the morphospecies level [8,9]. However, the overlapping distribution of multiple divergent lineages is a striking phylogeographic pattern that is common to most of the organisms, and it raises the question of whether some of these lineages are in fact cryptic species. In the light of present global climate change, on a population level, it is necessary to assess the status of these divergent lineages and delimit evolutionarily significant units (ESUs) [9], which is the foundation of forecasting the influence of climate change on intraspecific genetic variation.

In the marine environment, ocean currents are believed to have a major influence on the population connectivity of marine species. The coastal water of Japan is characterized by a series of ocean currents. In the Pacific Ocean of the Japanese islands, two western boundary currents, the subtropical warm and saline Kuroshio Current and the subarctic cold and less saline Oyashio Current, meet each other off the east coasts of the islands. These two currents produce the mixed water mass region between the Kuroshio and Oyashio Fronts, where the most pronounced latitudinal gradients of the surface temperature and sea surface salinity are observed in the Northwestern Pacific [10]. Thus, these warm and cold ocean currents produce stable and distinct thermal environments along the latitude throughout the year. In the Sea of Japan, a branch of the warm Kuroshio Current, the Tsushima Current, is the only warm current that flows along the Sea of Japan coast off of the Japanese islands. The Tsushima Current supplies a large quantity of heat from low to high latitudes and produces weakening thermal stress along the coasts of the Japanese islands [11].

The Japanese sand lance *Ammodytes personatus*, is a cold water species in the Northwestern Pacific. Populations of *A. personatus* are distributed along both sides of the coast around Japan and the Yellow Sea throughout subtropical and cold-temperate coastal areas, facing contrasting sea surface temperatures (i.e., the Kuroshio, Oyashio and Tsushima Currents), which implies an impressive tolerance to a broad range of temperatures. The species is sensitive to warm temperatures [12]; they bury themselves in sand when the water temperature increases to 17–20 °C during the summer in southern and central Japan populations [12]. Rather than a long migration, adults of *A. personatus* perform a microgeographic migration [13]. Clear genetic differences were found between populations north of the Iwate Prefecture and those south of the Miyagi Prefecture by isozyme and morphological analyses [13,14]. The boundary of the two groups was consistent with the transition zone between the Kuroshio and Oyashio Currents. However, different conclusions were inferred from the mitochondrial DNA (mtDNA results) [15]. Two highly divergent mitochondrial lineages (lineages A and B) were detected in *A. personatus*. The mtDNA lineage A was distributed in all populations from Hokkaido to the Yellow Sea, whereas the mtDNA lineage B was mainly found in the northern group. The distribution of haplotype lineage B was highly limited by the annual sea temperature [13].

The overlapping distribution of two divergent lineages in the northern and southern groups raises the question of whether these two lineages or the two geographic groups represent cryptic species. For any species, the success of conservation programs and the creation of effective management policies depend on determining the levels of genetic divergence within and between populations and on developing strategies to maintain genetic diversity. Evidence of whether a single species is present in the Northwestern Pacific Ocean or whether different significant evolutionary units appear in its distribution would be important in estimating the effect of global climate change on biodiversity. Bálint et al. showed that without correctly recognized intraspecific evolutionary significant units, the effects of global climate change are likely to be drastically underestimated [7]

MtDNA phylogeny represents only the genealogy of a single gene that is almost only maternally inherited. Therefore, the above questions could not be answered by employing mtDNA markers. Amplified fragment length polymorphism (AFLP) was introduced by Vos and colleagues in 1995 [16]. This technique can provide a very large number of polymorphic markers with a very fast and relatively simple laboratory procedure and is widely used for the identification of cryptic evolutionary units [17].

In this study, we evaluated a multilocus approach employing AFLP to investigate the genetic structures of *A. personatus* and compare the genetic variation of *A. personatus* in different ocean currents characterized by thermal environments. The status of independence of the different *A. personatus* mtDNA lineages was also estimated.

## 2. Materials and Methods 

### 2.1. Sample Collection

**Figure 1:**
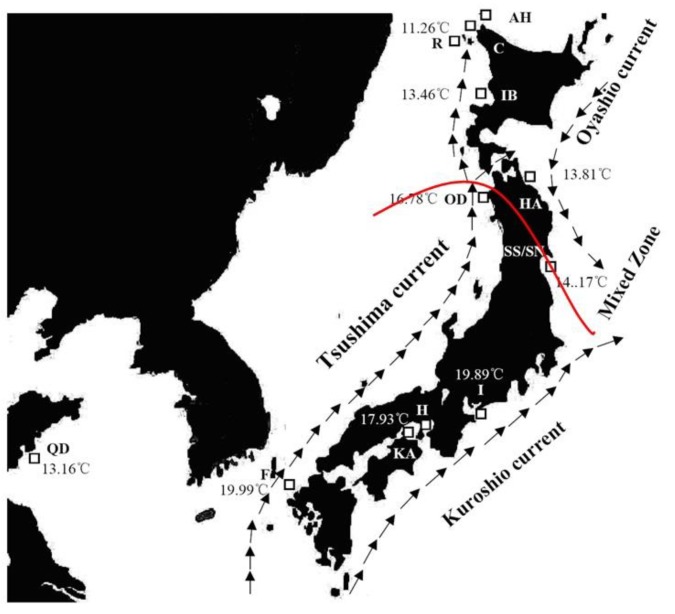
Map of the study area depicting sample locations, schematic map of currents, and the annual sea temperature. The samples ID in map are listed in Table 1. The predicted boundary between the northern and southern clades is represented on the map with a red line.

Two hundred and eleven individuals of *A. personatus* from twelve populations were collected throughout the species distribution (Table 1; Figure 1). The southern and northern groups were identified according to the descriptions of Okamoto et al. and Hashimoto and Kawasaki [14,18,19]. Two samples (SS, SN) belonging to the northern and southern groups were collected from the same location in Sendai Bay. Oceanographic features, including currents and annual sea surface temperatures (due to the lack of long migration in adults and vertical migration at night, the annual surface temperature was chosen), at the sampled localities are shown in Figure 1 and Table 1. The annual sea surface temperature from the Japan Oceanographic Data Centre) is shown in Figure 1 [20]. Thirty-seven individuals of *A. hexapterus* collected from Cape Soya, Japan served as the outgroup. Muscle samples were obtained and were either preserved in 95% ethanol or frozen for DNA extraction after specimen identification and morphological measurement. To estimate the status of independence of the different *A. personatus* mtDNA lineages, the mtDNA haplotype information was taken from our previous study [15]. The frequency of the two lineages for each population is given in Table 1.

### 2.2. AFLP Analysis

Genomic DNA was isolated from muscle tissue by proteinase K digestion followed by standard phenol–chloroform purification. DNA was subsequently resuspended in 100 μL of TE buffer (10 mmol/L Tris-Cl, 1 mmol/L EDTA, pH = 8.0). AFLP procedures were essentially based on Vos et al. and Wang et al. [16,21]. PCR products were resolved by 6.0% denaturing polyacrylamide gel electrophoresis (PAGE) for 2.5 h at 50 °C on a Sequi-Gen GT Sequencing Cell (Bio-Rad, Berkeley, CA, USA) and were ultimately detected using the silver staining technique. Sequences of AFLP adapters and primers are listed in Table 2. Four primer combinations (E-AAC/M-CTC, E-AAG/M-CAA, E-AAG/M-CAC and E-AGC/M-CAC) were chosen for AFLP analysis.

### 2.3. Data Analysis

Negative controls were run at each step of the AFLP process to check for exogenous contaminations. Samples with poor DNA quality, such as those with fragmented genomic DNA on agarose gel, were not used. Individuals with odd profiles, in which most of the bands were not observed in other individuals, were discarded. Bands with rare alleles (less than 4 individuals present) were also discarded.

AFLP bands were scored for presence (1) or absence (0) and were transformed into a 0/1 binary character matrix. Based on the binary matrix, genotypes, percentage of polymorphic bands, similarity indices, and genetic distance were obtained. Similarity indices were calculated using the formula S = 2N_ab_/ (N_a_ + N_b_) [22], where N_a_ and N_b_ are the number of bands in individuals a and b, respectively, and N_ab_ is the number of shared bands. Genetic distances between individuals were computed using the formula D = −ln S. For the calculation of the above values, we used a Microsoft Excel-based BASIC program (AFLP Data Analyser v1.3, city, country). The proportion of polymorphic loci, Nei’s genetic diversity, and Shannon diversity index were calculated using POPGEN (University of Alberta, Edmonton, Canada). The spatial genetic structure was examined using the Bayesian assignment probability test in the program STRUCTURE 2.3 (Stanford University, Palo Alto, CA, USA) [23]. This program uses a Bayesian approach to generate posterior probabilities of assignment of individuals to each of a given number of groups. The traditional Bayesian approach using software STRUCTURE (Stanford University, Palo Alto, CA, USA) for the evaluation of genetic population structure only highlights the main genetic structure of studied populations at a wide scale. In order to identify hierarchical and more detailed genetic structure, a multistep approach has been used, which entails a separate re-analysis of the groups identified in the previous steps [24]. In this study, we adopted the multistep approach that consists of running STRUCTURE a first time in order to identify a main structure and then re-running the software separately for two clusters identified in the previous step. At each step, we ran STRUCTURE according to admixture model, using a burnin period of 20,000 followed by 200,000 Markov chain Monte Carlo (MCMC). The procedure of AFLP markers analysis in the program STRUCTURE was followed Falush et al. [25]. The best k value was calculated from the online software Structure Harvester (http://taylor0.biology.ucla.edu/struct_harvest/). The genetic relationship between populations was estimated by constructing the Neighbor joining (NJ) tree based on Nei’s 1972 original distance in Mega 6.0 (Tokyo Metropolitan University, Tokyo, Japan) and evaluated with 1000 bootstrap replicates [26]. The NJ tree of all individuals was also constructed in Mega 6.0. In order to reveal the possible hybrid individuals between two clades, principal component analysis (PCA) was conducted in NTSYSpc [27]. The population structure was investigated using the analysis of molecular variance (AMOVA) and *F*-statistics in ARLEQUIN 3.5 (University of Bern, Bern, Switzerland) [28].

A plot figure was made to infer the relationship between the sampling latitude and the annual temperature. To further link genetic differentiation with annual sea surface temperature and geographic distance, which might influence the observed genetic structure at different spatial scales, the association between genetic differentiation (*F*_ST_) and pairwise differences in sea surface temperature, and isolation by distance (IBD) were tested using the Mantel test in ARLEQUIN 3.5 (University of Bern, Bern, Switzerland). Considering the two-dimensional habitats of *A. personatus* (along both coasts of Japan), pairwise values of log *F*_ST_ were plotted against logarithm of distance (two-dimensional stepping-stone model).

## 3. Results

Four AFLP primer combinations yielded clear bands and amplified a total of 272 loci across two species, of which 179 were polymorphic, with the primer combination E-AAC/M-CTC amplifying the largest number of loci, and primer combination E-AGC/M-CAC amplifying the lowest number of loci.

**Figure 2:**
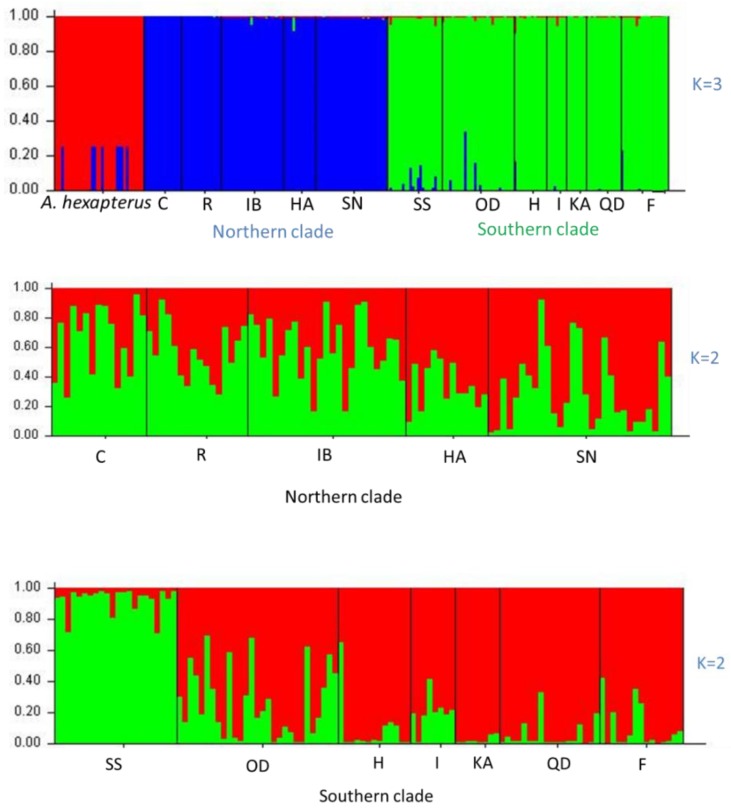
Spatial genetic structure for all individuals and northern and southern clades according to a Bayesian assignment probability analysis using the program STRUCTURE 2.3. K = 3 for the total data. K = 2 for northern and southern clades appeared to be the optimal number of clusters by showing the greatest ΔK. The samples ID are listed in Table 1.

The first step of Bayesian population assignment analysis for all 247 individuals using the software STRUCTURE revealed three clades (labelled southern clade, northern clade, and *A. hexapterus*) (Figure 2). In the second step, we re-ran a population structure analysis separately for the identified southern and northern clades in the first step. The presence of 2 clusters (K = 2) in the southern clade were revealed by the admixture model, identifying population SS from Sendai Bay as a single cluster (Q_p_ =92.4%) and all other populations pertaining to a second cluster (Q_p_ always higher than 74%). The analysis of the northern clade also identified two clusters. The first cluster dominated the populations from Cape Soya, Rebun Island, and Ishikari Bay (Q_p_ varied from 59.3% to 65.7%). The second cluster dominated the populations from Sendai Bay and Hachinohe (Q_p_ varied from 65.6% to 69.6%).

**Figure 3:**
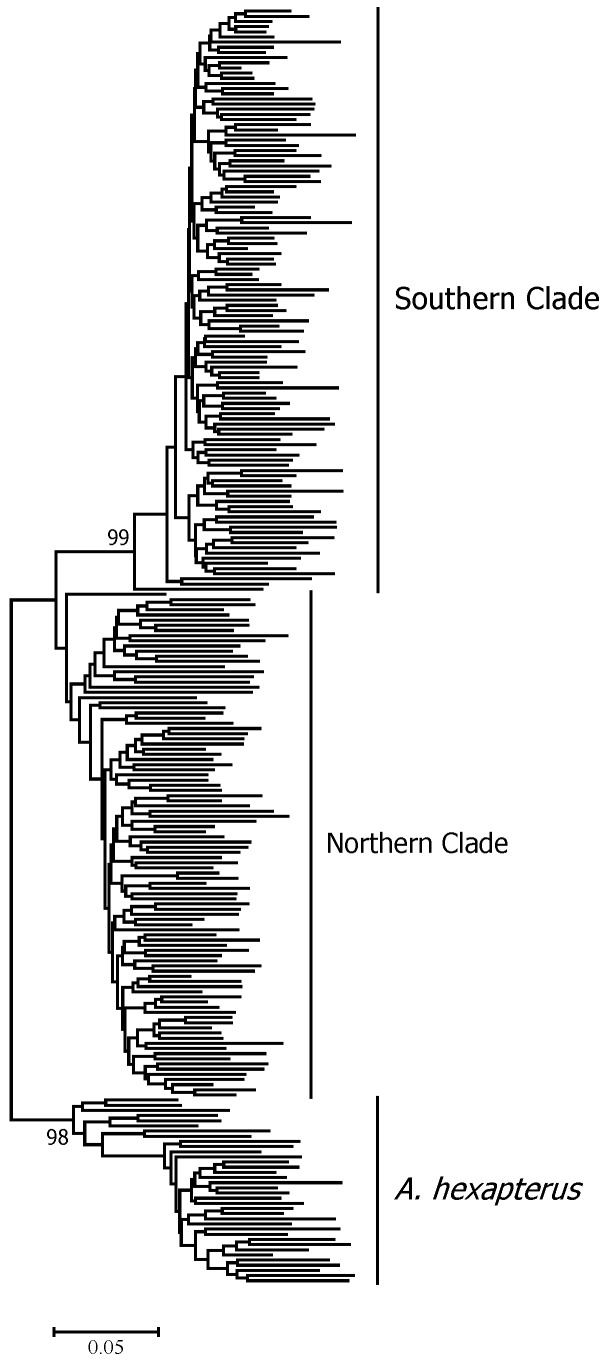
NJ tree based on Nei and Li’s (1979) genetic distances among all *Ammodytes* individuals [22]. Bootstrap supports of >90% in 1000 replicates are shown.

**Figure 4:**
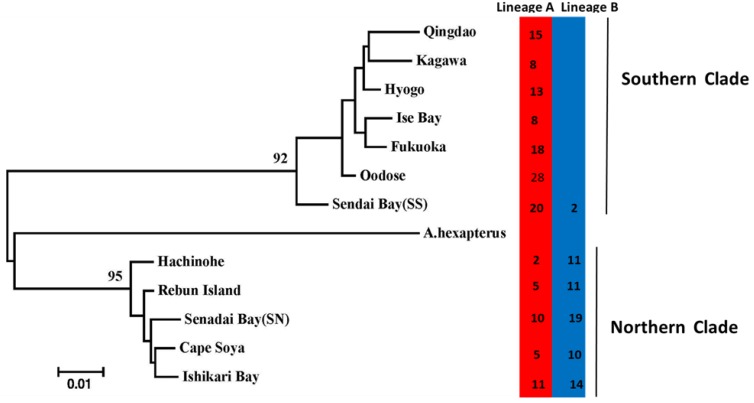
NJ tree based on Nei’s (1972) original genetic distances among populations [26]. The mtDNA lineages for each population were calculated from Han et al. [15]. Bootstrap supports of > 90% in 1000 replicates are shown.

**Figure 5:**
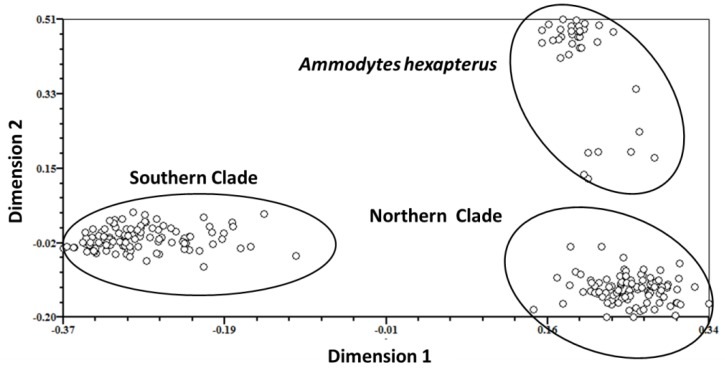
Two-dimensional scaling of two clades by principal component analysis (PCA) using AFLP markers.

One cluster analysis of all individuals was performed on all of the data obtained from four primer pairs. The results are illustrated as a dendrogram in Figure 3, in which three clades were confirmed. In this dendrogram, *A. personatus* and *A. hexapterus* are completely separate. Evidence from Bayesian population assignment analysis and the NJ tree indicated the existence of two clearly distinguishable clades (southern and northern clades) in *A. personatus*, which were sympatric within Sendai Bay. The separation of southern and northern clades was consistent with the definition of southern and northern groups based on previous allozyme data. Several specific bands were found among the three clades. The NJ tree of 13 populations also showed the complete divergence of northern and southern clades (Figure 4). Of the 211 individuals, 98 from four populations belonged to the northern clade, and 113 from seven populations belonged to the southern clade (Table 1). PCA analysis of the similarity data generated from the data of all five primer combinations (Figure 5) produced a picture similar to the results given by the Bayesian population assignment analysis and cluster analysis (Figure 2 and Figure 3), which revealed no hybrid individuals. The average genetic distances between clades were northern clade/southern clade: 0.1197; northern clade/*A. hexapterus*: 0.2275; and southern clade/*A. hexapterus*: 0.2586.

In the southern clade, the OD population had the highest proportion of polymorphic loci (58.48%) and number of polymorphic loci (131), whereas the KA population had the lowest number of polymorphic loci (42) and a proportion of polymorphic loci of 27.27% (Table 3). The proportion of polymorphic loci varied greatly in the northern clade, ranging from 48.98% to 75.43%. In general, the proportion of polymorphic loci in the northern clade was higher than that in the southern clade. The Nei’s genetic diversity and Shannon diversity index varied greatly in populations of *A. personatus*, ranging from 0.0641 to 0.1531 and 0.1042 to 0.2527, respectively. Thus, there were distinct variations among the 12 populations. Compared with populations of *A. personatus*, the population of *A. hexapterus* from Cape Soya showed a higher proportion of polymorphic loci and higher Nei’s genetic diversity and Shannon diversity index.

There were strong geographic differences in the distributions of the two clades (Figure 2). The southern clade consisted of seven populations and dominated the southern part of the Japanese coast and the Yellow Sea. Oodose in the Japan Sea and Sendai Bay in the Pacific Ocean could represent the northern boundary of the southern clade (Figure 4). The northern clade consisted of five populations and dominated the northern part of the Japanese coast. Sendai Ba in the Pacific Ocean was the southern boundary of the northern clade. The northern and southern clades coexisted in Sendai Bay, consistent with previous isozyme data. However, the northern and southern clades did not represent the mtDNA lineage A and B, respectively. Lineage A had a more extensive geographical distribution and was present in all 12 populations from Cape Soya to Qingdao (Table 1, Figure 4). It dominated the southern clade. Lineage B was sympatric with lineage A in seven populations, including two populations in the southern clade and all populations in the northern clade. Combination of AFLP and mtDNA data, the northern clade was originated from secondary populations of two mtDNA lineages. There was complete genetic isolation between northern and southern clades based on the nuclear makers, which represented two new species.

The genetic break between the northern and southern clades was also supported by AMOVA, with 44.17% of all variance partitioned between the northern and southern clades (*F*_CT_ = 0.4417, *p* < 0.001). Populations within the northern and southern clades also showed significant geographic structures, which were consistent with the subtle structure identified by the second step of Bayesian clustering analysis performed in southern and northern clades. Within the southern clade, 12.71% of genetic variance was attributed to differences between populations (*F*_ST_ = 0.1271, *p* < 0.001). Within the northern clade, 4.56% of the variance was attributed to differences between populations (*F*_ST_ = 0.0456, *p* < 0.001). Similar results were also revealed in pairwise *F*_ST_ values. All pairwise *F*_ST_ values were high and significant except for the *F*_ST_ values between Cape Soya and Rebun Island. Within the southern clade, *F*_ST_ values among populations ranged from 0.0573 to 0.2078 (Table 4). Similarly, pairwise *F*_ST_ values among populations in the northern clade ranged from 0.0112 to 0.0791.

**Figure 6:**
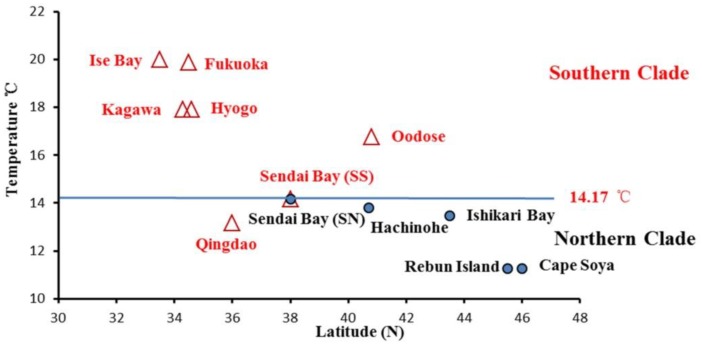
The relationship between the sampling latitude and the annual surface temperature of 12 populations. Triangles represent southern clade populations, circles represent northern clade populations.

**Figure 7:**
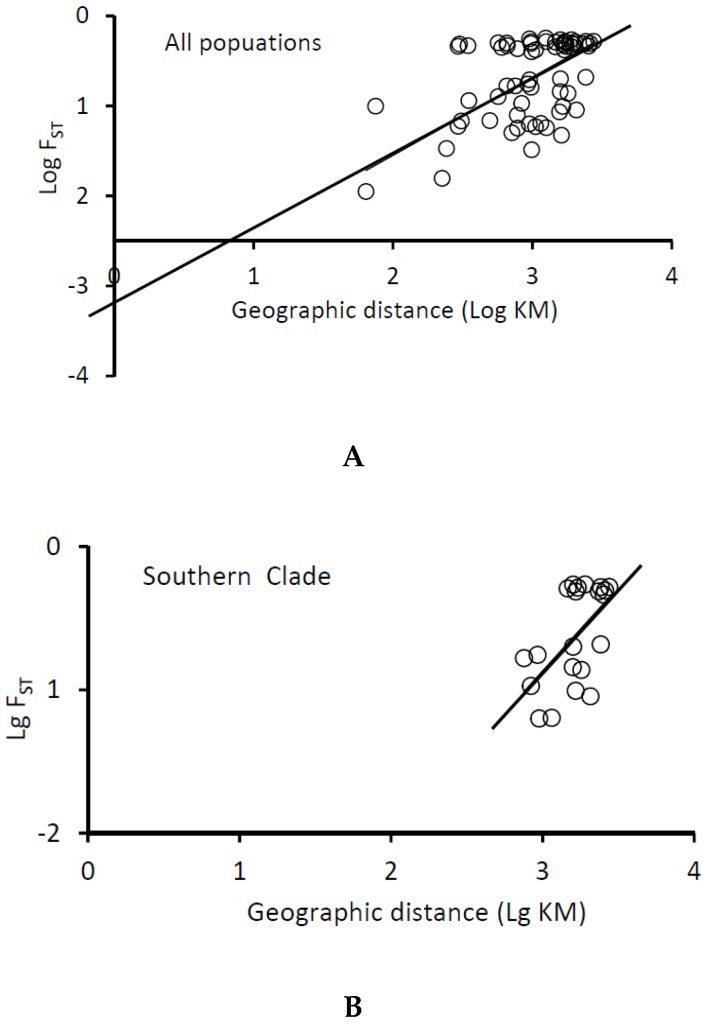

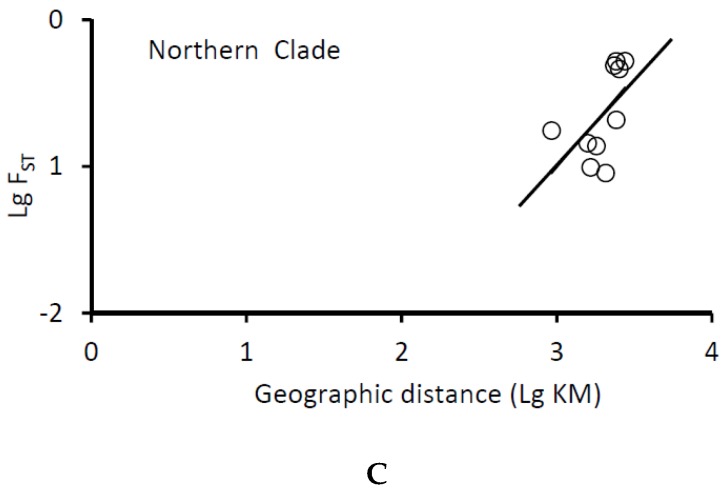
Plot of pairwise estimates of Log *F*_ST_ vs. geographic distance (Log KM) between populations. (**A**): all populations (**B**): southern clade populations (**C**): northern clade populations.

The annual sea temperatures for the southern and northern clades varied from 13.16 °C to 19.99 °C and 11.26 °C to 14.17 °C, respectively (Figure 1). The plot figure for the relationship between the latitude and annual sea temperature of populations excluding Qingdao showed the obvious separation of the northern and southern clades by annual sea temperature (Figure 6). The separation annual temperature between northern and southern clades were 14.17 °C. For the mantel test, pairwise temperature and geographic distance were considered as major factors significantly associated with genetic differentiation in the entire study area (Table 5, Figure 7). In the southern clade, temperature was also significantly correlated with genetic differentiation, with temperature variation explaining 46% of the variation in genetic differentiation in this clade (*p* = 0.02). However, there was no evidence of isolation by distance in the southern clade, with geographic distance explaining very little of the variation (r = 0.14, *p* = 0.35) (Table 5). In the northern clades, Mantel test indicated a significant relationship (*p* = 0.02) between Log ***F*_ST_** and geographic distance (Log km) indicating isolation by distance, with geographic distance explaining 76% of the variation in genetic differentiation for the northern clade (r = 0.76). However, the matrix correlation explained very little of the variation (r = 0.04) and there was no significant relationship between pairwise genetic differentiation and sea surface temperature in the northern clade (*p* = 0.33).

## 4. Discussion

The genetic variation of the population of *A*. *personatus* around the Japanese islands has been studied by morphology, isozymes, and mtDNA sequences [14,15,18,19]. Morphological population studies of *A*. *personatus* in the context of the vertebral count revealed that two groups with a vertebral count mode of approximately 65 or 63 inhabit the seas around Japan, and they intermingle in Sendai Bay off Aomori and Miyagi [18]. In a previous isozyme study, *A. personatus* distributed around Japan consisted of two populations: the northern and southern populations [19]. However, no fixed locus was found between the two populations by isozyme analysis, and it was difficult to identify two populations at the individual level according to isozyme data. MtDNA control region sequence analysis of *A. personatus* revealed two distinct mtDNA lineages in the Northwestern Pacific, reflecting population isolation during low Pleistocene sea level stands [13]. Because mtDNA is maternally inherited, it is not possible to determine whether the presence of divergent mitochondrial lineages in the same population is a result of secondary contact after an extended period of isolation and/or the presence of two sibling species. In the present study, two completely isolated clades of *A. personatus* were revealed by AFLP markers, which were consistent with previous morphological and isozyme studies [14,18,19]. Several fixed loci were detected between the northern and southern clades. Considering the sympatry of the two clades in Sendai Bay, complete reproductive isolation may exist between these two clades. Although the incongruence between nuclear clades and mitochondrial lineages suggests that the two distinct mtDNA lineages do not represent cryptic species, the present study demonstrates that *A. personatus* is indeed composed of at least two genetically divergent new species in the Northwestern Pacific. The same lineage in the southern and northern clades belongs to different cryptic species, which revealed a historical mtDNA introgression from southern populations to northern populations.

Sharp phylogeographical breaks in all species indicate limits to dispersal over both contemporary and historical time scales [29]. Many authors suggest that Pleistocene glaciations were the most significant historical factors to shape the phylogeographic patterns and population structure in marine fish species [30,31]. Toews and Brelsford examined 126 cases in animal systems including 24 cases in fish where there was strong evidence of discordance between the biogeographic patterns identified in mitochondrial DNA and those observed in the nuclear genome [32]. The majority of cases (97%) were those where discordance likely arose following geographic isolation and secondary contact. Regions of mito-nuclear discordance are most apparent in northern groups of *A. personatus.* According to the general interpretation from Toews and Brelsford, the significant discordance between mtDNA and nuclear markers suggested *A. personatus* experienced at least two isolation events during Pleistocene glaciations. Lineage A and B dominated the south group and north group, respectively, indicating Pacific Ocean and Sea of Japan origin. The first isolation event created the two mtDNA lineages, which happened about 453,000 years ago during the separation of Pacific Ocean and Sea of Japan [15]. The two lineages began secondary contact in northern groups during the following postglacial period. Once again, *A*. *personatus* suffered long time isolation after secondary contact during another glacial period. During the second period of isolation, it is assumed that divergent northern and southern groups accumulated mutations in their nuclear genomes and developed reproductive isolation. Besides historical factors, intra- and interspecific genetic variation patterns are also greatly influenced by established barriers to marine animal movement created by circulation patterns, temperature regimens, and coastal topography [33]. In the present study, marked geographic structuring in *A. personatus* diverged into two genetically distinct new species (northern and southern). The distributional range of the two species appears mainly to be shaped by the major oceanographic current systems. A candidate for a barrier to gene flow between the northern and southern clades of *A. personatus* is the temperature difference of the sea water derived by the Kuroshio and Oyashio Currents.

On the Pacific side of the Japanese islands, the boundary of the two clades was consistent with the transition zone of the Kuroshio and Oyashio Currents. The Kuroshio Current flows along the southern coast of the Japanese Islands and towards the east off central Japan as a part of the wind-driven subtropical gyre circulation cell. The Oyashio Current is the western component of the Kamchatka–Alaskan Current and carries cold and high-nutrient surface water to the Northwestern Pacific. The great sea surface temperature gradient between the Kuroshio and Oyashio Currents may function as an effective physical barrier between the northern and southern clades.

According to the mantel test, pairwise temperature was considered a major factor significantly associated with genetic differentiation for the entire study area and southern clade. Such close relationships between sea surface temperature and clade distributions presumably reflect possible temperature adaptions. The annual temperature ranged from 13.16 to 19.99 °C in the southern clade samples, with a relatively high temperature; the northern clade sampling waters were characterized by lower temperature, which ranged from 11.26 to 14.17 °C (Figure 1, Figure 4). Different performances of mantel tests in both clades were revealed, indicating significant isolation by surface temperature in the southern clade and non-significant isolation by surface temperature in the northern clade. This suggested that environmental divergence had a larger impact on genetic divergence in the southern clade than in the northern clade. Isolation by surface temperature in the southern clade indicated that populations of the southern clade were adapted to local surface temperature. Absence of isolation by surface temperature in the northern clade indicated these populations were in the optimum range of temperature. An interesting result was found in the Qingdao population from the southern group with a relatively low annual sea surface temperature (13.16 °C), which was a lower temperature than the northern margin population of Sendai in the southern group. Competitive release may be a possible explanation for the distribution of the southern clade in the Yellow Sea. Without the competition of the northern clade in the Yellow Sea, the southern clade with wide temperature tolerance expanded its range and entered the Yellow Sea. Thus, the northern and southern clades might have different temperature adaptations. The northern clade belonged to the stenothermal type, which was restricted by high sea temperature (the annual sea temperature of 14.21 °C might be the threshold temperature). Although local adaption was revealed, the southern clade might fit a wide range of temperatures; the low threshold temperature may even be below 13.16 °C. The eurythermal character of the southern clade and the cold bias character of the northern clade was evidenced by our previous mtDNA results [15]. The mtDNA lineages A and B represent the eurythermal and cold stenothermal types, respectively. The southern clade contained lineage A in all populations and a very low percentage of lineage B in the transition zone (only 11.3 %). However, the northern clade contained a low frequency of lineage A (17.1% to 43.5% among different populations) and a high frequency of lineage B (56.5% to 82.9% among different populations) [15].

Local temperature adaption was reported in some marine fishes. The distributional range of the three cryptic species of *M. cephalus* in the Northwestern Pacific was associated with different surface temperatures, indicating local thermal adaptation [34]. The simple sequence repeat (SSR) results of Atlantic salmon, *Salmo salar*, suggested that the adaptation of the thermal regime mainly explained the hierarchical genetic structure observed among salmon populations [35]. Santos et al. also suggested that differences in seawater temperature in the tropical and subtropical regions represented the main factor that isolated the *Macrodon* groups [36].

Besides temperature, isolation by distance was also the important mechanism that promoted genetic differentiation in *A. personatus*, especially within the northern clade. The plot of Log *F*_ST_ and geographic distance (Log KM) revealed a strong pattern of isolation by distance in the northern clade, explaining about 76% of the variation in gene flow. Isolation by distance in the northern clade indicates that this clade is at genetic equilibrium under dispersal and genetic drift [37]. In contrast, the southern clade did not exhibit a pattern of isolation by distance. Absence of isolation by distance may be caused by insufficient time following a recent range expansion in the southern clade. Considering the Holarctic origin for *Ammodytes* species, it is reasonable that the southern clade colonized its current habitat recently.

The correlation between the sea temperature and genetic structure of marine organisms identified temperature-linked spatial distributions in some evolutionary units, which helped us to predict the species distribution shift under different scenarios of global climate change. Environmental niche models have been widely used to predict the distributions of plants and animals [38], which are based on nominal morphospecies and ignore intraspecific genetic diversity, biotic interactions, and species dispersal [1]. A bioclimate envelope can be defined as a set of physical and biological conditions that are suitable for a given species. Thus, shifts in species distributions can be predicted by evaluating changes in bioclimate envelopes under climate change scenarios [38]. However, there are some fundamental shortcomings. It is clear that the cryptic diversity affects global climate change projections [1]. In a recent study, Bálint et al. showed that without discerning intraspecific genetic variation and cryptic diversity, the effects of global climate change were likely to be drastically underestimated [7]. From our findings, the southern and northern clades of *A. personatus* may represent two cryptic species in *A. personatus* with different and independent significant evolutionary units and different thermal adaptations. Furthermore, competitive release and competitive exclusion among cryptic species may cause error in estimating the preferred temperature for species. If the present study was only conducted in the coastal waters of the Japanese islands, uncovering the Yellow Sea, 14.21 °C was the lower boundary of annual temperature for the southern group. Based on this temperature value, the absence of the southern group in the northern coast of the Japanese islands was likely because the cold temperature limited the distribution of the southern group to the north. Competitive release and competitive exclusion were revealed, which may explain the distributions of the two groups. Thus, our results demonstrate that cryptic diversity should be considered when estimating the effects of climate change on biodiversity.

## 5. Conclusions

The present study successfully identified two cryptic *A. personatus* species in the Northwestern Pacific Ocean by neutral AFLP markers. The genetic patterns of the two clades of *A. personatus* were greatly influenced by Pleistocene glaciation isolations, the local sea temperature, and isolation by distance. The distribution of the northern clade may be a particularly good indicator of sea surface temperature change. Under the following climatic warming scenario, the distribution of the northern group may become more restricted to high latitudes. The southern group is likely to be less dramatically affected by climate change and to maintain the stable southern boundary by summer aestivation and a shift of the northern boundary to high latitudes. Our results demonstrate that intra-species diversity and competitive release between significant evolutionary units should be taken into account to assess the influence of climate change on species. In future studies, the exact range of temperature in both groups must be assessed with a sufficient number of samples to predict the influences of global climate change on both groups.

## Figures and Tables

**Table 1:** Sampling information, number of genotypes based on Amplified fragment length polymorphism (AFLP) and allozyme analyses, and types of mtDNA lineages.

Species	ID	Sample Location	Sample Size	Date of Collection	Genotype Based on AFLP	Genotype Based on Allozyme *	mtDNA lineage *
*A. personatus*	QD	Qingdao	15	April 2005	Southern clade	Southern group	One lineage(A)
	F	Fukuoka	18	April 2005	Southern clade	Southern group	One lineage(A)
	KA	Kagawa	8	April 2005	Southern clade	Southern group	One lineage(A)
	H	Hyogo	13	April 2005	Southern clade	Southern group	One lineage(A)
	I	Ise Bay	8	May 2005	Southern clade	Southern group	One lineage(A)
	OD	Oodose	29	May 2006	Southern clade	Southern group	Two lineages (A: B = 28:1)
	SS	Sendai Bay	22	April 2006	Southern clade	Southern group	Two lineages (A: B = 20:2)
	SN	Sendai Bay	29	April 2006	Northern clade	Northern group	Two lineages (A: B = 10:19)
	HA	Hachinohe	13	June 2005	Northern clade	Northern group	Two lineages (A: B = 2:11)
	IB	Ishikari Bay	25	April 2006	Northern clade	Northern group	Two lineages (A: B = 11:14)
	R	Rebun Island	16	June 2006	Northern clade	Northern group	Two lineages (A: B = 5:11)
	C	Cape Soya	15	June 2006	Northern clade	Northern group	Two lineages (A: B = 5:10)
*A. hexapterus*	AH	Cape Soya	36	June 2006			
Total			247				

* Genotypes based on allozyme and mtDNA lineage analyses according to Han et al [15].

**Table 2:** Adaptor and primer sequences used in the AFLP analysis.

Primer	Sequence
Adapters	
*EcoR*I adapter	5’-CTCGTAGACTGCGTACC-3’
	5’-AATTGGTACGCAGTCTAC-3’
*Mse*I adapter	5’-GACGTGAGTCCTGAG-3’
	5’-TACTCAGGACTCAT-3’
Pre-amplification primer	
*Eco*RI	5’-GACTGCGTACCAATTC-3’
*Mse*I	5’-GATGAGTCCTGAGTAA-3’
Selective amplification primer	
E-AAC/M-CTC	5’-GACTGCGTACCAATTCAAC-3’ 5’-GATGAGTCCTGAGTAACTC-3’
E-AAG/M-CAA	5’-GACTGCGTACCAATTCAAG-3’ 5’-GATGAGTCCTGAGTAACAA-3’
E-AAG/M-CAC	5’-GACTGCGTACCAATTCAAG-3’ 5’-GATGAGTCCTGAGTAACAC-3’
E-AGC/M-CAC	5’-GACTGCGTACCAATTCAGC-3’ 5’-GATGAGTCCTGAGTAACAC-3’

**Table 3:** Genetic diversity analyses of 12 *Ammodytes personatus* populations and one *Ammodytes hexapterus* population.

	QD	F	KA	H	I	SS	SN	OD	HA	IB	R	C	AH
Number of loci	188	189	154	183	173	215	228	224	196	231	175	211	245
Percentage of polymorphic loci	49.47%	44.97%	27.27%	44.81%	39.88%	54.42%	57.02%	58.48%	48.98%	56.71%	75.43%	54.98%	62.45%
Nei’s genetic diversity	0.1081	0.0794	0.0641	0.0907	0.0958	0.1029	0.0932	0.0982	0.0944	0.1047	0.1531	0.1263	0.1218
Shannon diversity index	0.1772	0.1350	0.1042	0.1506	0.1555	0.1711	0.1578	0.1667	0.1592	0.1750	0.2527	0.2035	0.2011

**Table 4:** Pairwise *F*_ST_ values among 13 *Ammodytes* populations.

	QD	F	H	KA	I	OD	SS	SN	HA	IB	R	C
F	0.1757*											
H	0.0986*	0.1066*										
KA	0.1440*	0.1665*	0.0993*									
I	0.1378*	0.0632*	0.0679*	0.1143*								
OD	0.0901*	0.0638*	0.0473*	0.0857*	0.0573*							
SS	0.2078*	0.2003*	0.1606*	0.1951*	0.1666*	0.1269*						
SN	0.5222*	0.5430*	0.5135*	0.5549*	0.4991*	0.5016*	0.4652*					
HA	0.5223*	0.5442*	0.5176*	0.5669*	0.4931*	0.4860*	0.4596*	0.0594*				
IB	0.4884*	0.5105*	0.4770*	0.5098*	0.4521*	0.4661*	0.4316*	0.0566*	0.0685*			
R	0.4623*	0.4865*	0.4545*	0.4844*	0.4194*	0.4461*	0.3998*	0.0326*	0.0503*	0.0157*		
C	0.4945*	0.5161*	0.4902*	0.5144*	0.4570*	0.4670*	0.4201*	0.0588*	0.0791*	0.0336*	0.0112	
AH	0.5547*	0.5718*	0.5356*	0.5724*	0.5386*	0.5397*	0.5263*	0.4613*	0.4471*	0.4539*	0.4256*	0.4472*

*, significant at *p* < 0.05 according to the permutation test.

**Table 5:** Results of Mantel tests (*F*_ST_ vs. temperature, Log *F*_ST_ vs. geographic distance Log KM) at the different spatial scales.

Isolation by Temperature	Mantel test	All Populations	Northern Clade	Southern Clade
Isolation by distance	r	0.41	0.04	0.46
	*P*	0.02	0.33	0.02
	*r*	0.26	0.76	0.14
	*P*	0.02	0.02	0.35
